# Neuroprotective and Anti-inflammatory Role of Atorvastatin and Its Interaction with Nitric Oxide (NO) in Chronic Constriction Injury-induced Neuropathic Pain

**DOI:** 10.22037/ijpr.2020.1101230

**Published:** 2020

**Authors:** Amin Hasanvand, Fariba Ahmadizar, Abolfazl Abbaszadeh, Ahmad-Reza Dehpour, Hossein Amini-khoei, Amir Abbasnezhad, Ali Kharazmkia

**Affiliations:** a *Department of Physiology and Pharmacology, Faculty of Medicine, Lorestan University of Medical Sciences, Khorramabad, Iran. *; b *Department of Epidemiology, Erasmus University Medical Center, Rotterdam, The Netherlands. *; c *Department of Plastic and Reconstructive Surgery, Hazrat Fatemeh Hospital, School of Medicine, Iran University of Medical Sciences, Tehran, Iran. *; d *Department of Pharmacology, School of Medicine, Tehran University of Medical Sciences, Tehran, Iran. *; e *Experimental Medicine Research Center, Tehran University of Medical Sciences, Tehran, Iran.*; f *Medical Plants Research Center, Basic Health Sciences Institute, Shahrekord University of Medical Sciences, Shahrekord, Iran. *; g *Nutritional Health Research Center, Lorestan University of Medical Sciences, Khorramabad, Iran. *; h *Department of Clinical Pharmacy, Faculty of Pharmacy, Lorestan University of Medical Sciences, Khorramabad, Iran.*

**Keywords:** Neuropathic pain, Nitric oxide, Chronic Constriction injury, Atorvastatin, Anti-inflammatory

## Abstract

Prevention and treatment of neuropathic pain (NP) is one of the most difficult problems in clinical practice since the underlying mechanism of NP is unclear. In previous studies, the increased production of nitric oxide (NO) has been closely linked to the induced NP. In this study, we assessed the effect of atorvastatin through NO mechanism, on inflammation, thermal hyperalgesia, thermal allodynia, and mechanical allodynia as well as sciatic nerve histological score in rat with chronic constriction injury (CCI) model. Finally, we specified the role of cytokines such as TNF-α and IL-6 in the spinal cord. Treatment with atorvastatin and L-NAME (NO inhibitor) attenuated the thermal hyperalgesia, thermal allodynia and mechanical allodynia induced by CCI. The antinociceptive consequence was better elevated with a combination of atorvastatin and L-NAME in comparison with the other groups. In addition, the treatment with these drugs also attenuated the CCI-induced TNF-α and IL-6 level in the spinal cord. Furthermore, the histological analysis showed a low level of inflammation in the sciatic nerve in the CCI rats co-treated with atorvastatin and L-NAME. Findings of our study in NP-induced CCI in the rat model demonstrate that inhibition of NO displays antinociceptive and anti-neuroinflammatory effects of atorvastatin in peripheral and central nervous system. In addition, we found that inhibition of the NO by atorvastatin could be one of the most important anti-inflammatory pathways of atorvastatin effect.

## Introduction

Neurological disorders in peripheral/central nervous system are normally induced by the impairment of the somatosensory functions (1). Neuropathic pain (NP) involves activation of the nociceptive pathways and unusual responses to any noxious irritant (hyperalgesia) or any innocuous irritant (allodynia) (2). Several pro-inflammatory cytokines, *e.g.* cytokine tumor necrosis factor (TNF)-α and interleukin 6 (IL-6), have been associated with the development of NP in various animal models (3). TNF-α has been considered as an original mediator in pro-inflammatory processes, therefore, it is one of the most important factors that cause NP (4, 5). Previous studies also reported the remarkable effect of IL-6 on the occurrence of NP (6). Increased IL-6 as a pro-inflammatory cytokine leads to induced allodynia and/or hyperalgesia due to nerve damage (7). 

Statins (HMG-CoA reductase inhibitors) are mainly well known for their anti-inflammatory and/or antioxidant impacts in many diseases including arthritis, cancer, and Alzheimer disease (8). Studies have revealed that atorvastatin use is associated with anti-inflammatory effects (9). Recently, Chu *et al.* have shown that atorvastatin use prohibits the progress of thermal hyperalgesia, mechanical allodynia, and neuroinflammation in Rats (10). 

Nitric oxide (NO) is considered as a major messenger to mediate neuronal and inflammatory signaling pathways in numerous physiological processes (11). Up-regulation of NO has been closely associated with the induced NP (12). Moreover, NO plays an important key in the development of paw withdrawal latency (PWL) in response to thermal and cold allodynia (13). NO is stimulated by IKappaB, immunoreceptors, cytokines as well as chemokine, and readily crosses the blood brain barrier to induce central cytokine production (14). Nowadays, studies have revealed that activation of NO plays an important role in the mechanisms underlying exaggerated pain sensitivity in the spinal cord (15).

The aim of this study was to investigate the role of atorvastatin and its interaction with NO on mobility behaviors and histopathology of nerve and its functions in the CCI rat model.

## Experimental

*Animals*

Seventy male adult Sprague–Dawley rats weighing 240-260 g were prepared from Lorestan University of Medical Sciences, (Khorramabad, Iran). The animals were housed in a colony room with a 12:12 h light/ dark cycle at 21 ± 2 °C and free access to water and food ad libitum; whereas the animals were handled according to the Institutional Animal Care and Use Committee guidelines of Lorestan University of Medical Sciences.

*Study design*

The animals were randomly divided into seven experimental groups (n = 10 rats per group): 1: Sham-operated (Sham), 2: CCI vehicle-treated (CCI), 3: CCI + Gabapentin (50 mg/kg), 4: CCI+ atorvastatin (ATOR). (10 mg/kg), 5: CCI+ ATOR. (10 mg/kg) + L-NAME (5 mg/kg), 6: CCI+ ATOR. (10 mg/kg) + L-arginine (100 mg/kg).

Atorvastatin (oral gavage treatment), L-NAME (IP injection) and L-arginine (IP injection) were administered every day from the one day before surgery to the 14th day after ligation. L-NAME (NO inhibitor) and L-arginine (NO donor) were administered fifty minutes before atorvastatin administration. The behavioral tests were performed on the day before surgery (corresponding to the day CCI in sham-operated) and 14 days after CCI. Fourteen days after CCI, the rats were sacrificed and anaesthetized with diethyl ether and blood samples were collected from the jugular vein. Then, measurements of inflammatory cytokines were performed. Pentobarbital sodium was used to achieve anesthesia. All drugs were freshly prepared in distilled water (16).

*Surgery*

In short, anesthesia was achieved through the administration of pentobarbital Na (60 mg/kg). After anesthesia, sciatic nerve (left nerve) was exposed and four ligations were carried out around the nerve proximal to the trifurcation. The space between the two adjacent ligatures was 1 mm (17). In the sham group, similar surgical method was performed excluding the nerve ligation.

*Thermal Hyperalgesia (Hot Plate Test)*

Reaction latencies of animal to thermal hyperalgesia (IITC Plantar Analgesia Meter (IITC Life Science Inc., CA)) at 52 ± 2 °C in the form of the first sign of nociception, licking, flinching or jump reaction to avoid the heat were measured, as described elsewhere on the 4^th^, 7^th^, and 14^th^ days after CCI. In order to prevent any damage to the tissues, the device was set to turn off automatically after 15 sec (18).

*Thermal Allodynia (Acetone Test)*

In this test, the animals’ response to acetone (spray 100 μL of acetone) was noted in 20 s and it was scored according to Kukkar and Singh scale defined in four points: 0: no reflex; 1: quick stamp, flick or withdrawal of the paw; 2: repeated flicking or prolonged withdrawal; and 3: repeated flicking with licking of the paw. The score range was recorded from zero to nine (19).

*Mechanical Allodynia (Von Frey Test)*

To determine mechanical allodynia, we used the von Frey filaments (0.6, 1.0, 1.4, 2.0, 4.0, 6.0, 8.0, 10.0, 15.0, 26.0 and 60 g). In this test, we determined the thresholds to mechanical stimuli for the plantar surface of the hind paw on all days of testing (20).

*Measurement of Inflammatory Cytokines*

The spinal nerve samples were collected and supernatant was separated at 4000 g for 15 min. After the collection of supernatant, it was stored in freezer (-70 °C) until the evaluation of inflammatory cytokines (such as TNF-α and IL-6) using ELISA kit (Abcam, USA) (21).

*Histological Studies*

The sciatic nerve was isolated and fixed in 10% formalin. The next phase included the division of samples into 5 separate parts that were of 5 micrometers. The divided samples were then stained with hematoxylin and eosin. A histologist, blinded to the study, analyzed the slides using previously established scales for perineural inflammation (22).

*Statistical Analysis*

Mean ± standard error of the mean (SEM) was used for indicating the result. The data that were collected after the evaluation of the results of behavioral tests, were analyzed via the utilization of a two-way analysis of variance (ANOVA). Subsequently, biochemical experiments were also investigated using ANOVA via` Graph pad prism Version-5.0 software. A *p*-value ˂ 0.05 was considered statistically significant. 

## Results

*Evaluation of Anti-Hyperalgesia Effects*

**Figure 1:**
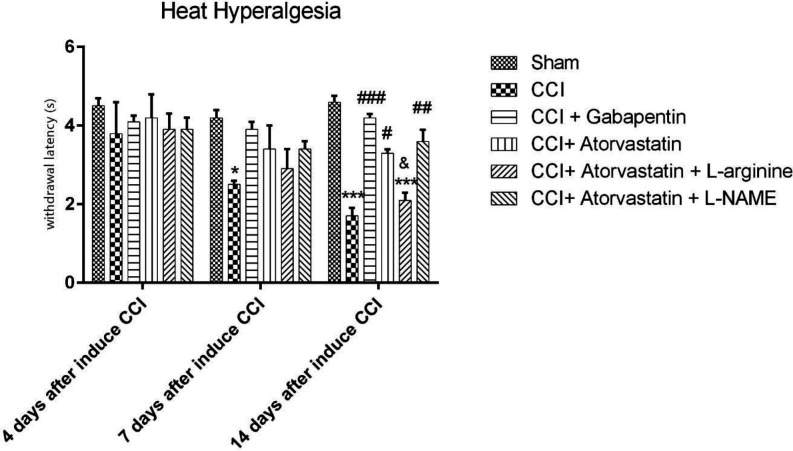
Effects of atorvastatin administration on CCI-induced heat hyperalgesia assessed by Hot Plate test (n = eight rats per each group). ^*^P < 0.05, ^**^P < 0.01, ^***^P < 0.001 vs. Sham, ^#^P < 0.05, ^##^P < 0.01, ^###^P < 0.001 vs. CCI, ^&^P < 0.05, ^&&^P < 0.01, ^&&&^P < 0.001 vs. CCI + ATOR + LOS

The CCI model significantly increased in response to hyperalgesia heat stimulation compared to the sham group on the 7^th^ and 14^th^ days of the study. Furthermore, there were significant distinctions in heat stimulation score in the CCI + ATOR and in the CCI + ATOR + L-NAME groups compared to the CCI group on the 14^th^ day (*P* < 0.05 and *P* < 0.01, respectively, Figure 1). 

The CCI rats treated with gabapentin for 14 days experienced the attenuation of CCI-induced hyperalgesia in NP (*P* < 0.001). Moreover, the co-administration of atorvastatin and L-NAME improved the thermal hyperalgesia on the 14^th^ day in comparison with group 6 (*P* < 0.05, Figure 1).

*Evaluation of Anti-Cold Allodynia Effects*

**Figure 2:**
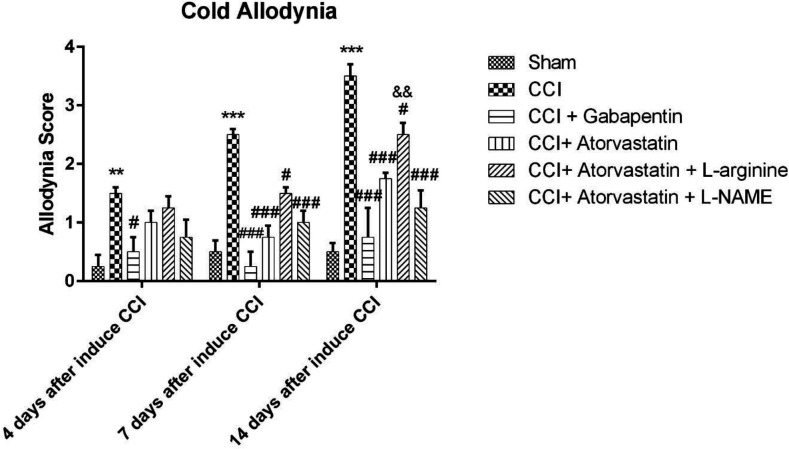
Effects of atorvastatin administration on CCI-induced cold allodynia assessed by acetone drop test (n = eight rats per each group). ^*^P < 0.05, ^**^P < 0.01, ^***^P < 0.001 *vs. *Sham, ^#^P < 0.05, ^##^*P* < 0.01*,* ^###^*P *< 0.001 *vs.* CCI, ^&^P < 0.05, ^&&^*P* < 0.01, ^&&&^*P* < 0.001 *vs.* CCI + ATOR + LOS

Figure 2 shows the rise in response to the stimulation due to the acetone spray on the rats’ feet, which were subjected to the imperfect ligation of the sciatic nerve, which entails the induction of NP among CCI rats. These differences were observed in the CCI group rather than in the sham group on the 4^th^, 7^th^ and 14^th^ days (*P *< 0.01, *P *< 0.001 and *P *< 0.001, respectively). Atorvastatin, atorvastatin + L-arginine and atorvastatin + L-NAME (*P *< 0.001, *P* < 0.05 and *P *< 0.001, respectively) successfully decreased the cold hypersensitivity in the CCI groups on the 7^th^ and 14^th^ days. The combination of atorvastatin + L-NAME more effectively reversed the cold allodynia, as compared to the group with atorvastatin + L-arginine. Furthermore, the co-administration of atorvastatin and L-NAME significantly improved the acetone drop on the 14^th^ day compared to the atorvastatin + L-arginine group (*P* < 0.01, Figure 2).

*Evaluation of Anti-Mechanical Allodynia Effects*

**Figure 3:**
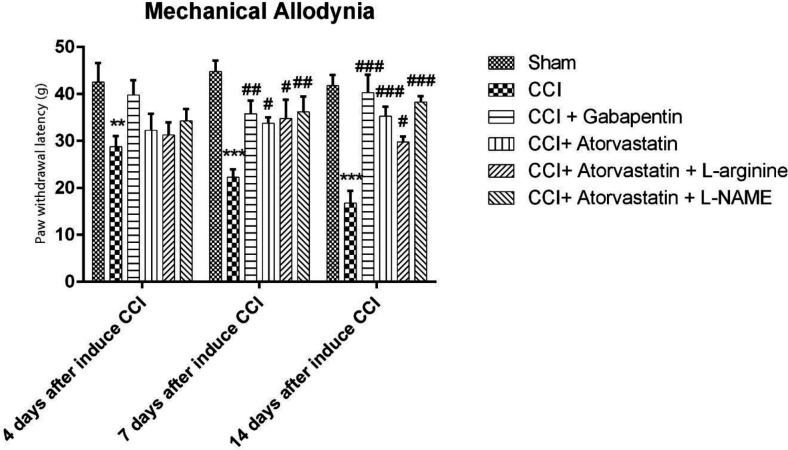
Effects of atorvastatin administration on CCI-induced mechanical allodynia assessed by von Frey hair test (n = eight rats per each group). ^*^P < 0.05, ^**^P < 0.01, ^*** ^*P* < 0.001 *vs.* Sham, ^#^P < 0.05, ^## ^*P < *0.01, ^### ^*P* < 0.001 *vs.* CCI, ^&^P < 0.05, ^&&^*P < *0.01, ^&&&^*P* < 0.001 *vs.* CCI + ATOR + LOS

Figure 3 indicates the results of the mechanical allodynia among the CCI rats (group 2). The obtained results showed that the PWL significantly decreased among the group 2. The PWL of the atorvastatin-treated group increased in the rats experienced with the CCI-induced NP (*P* < 0.001). Nonetheless, the positive effects of atorvastatin were reduced after the rats were treated with L-arginine (*P* < 0.05). However, the combination of atorvastatin with L-NAME further significantly increased the PWL in the CCI rats (*P* < 0.001).

*Inflammatory Cytokines Evaluation in Spinal Nerve*

**Figure 4:**
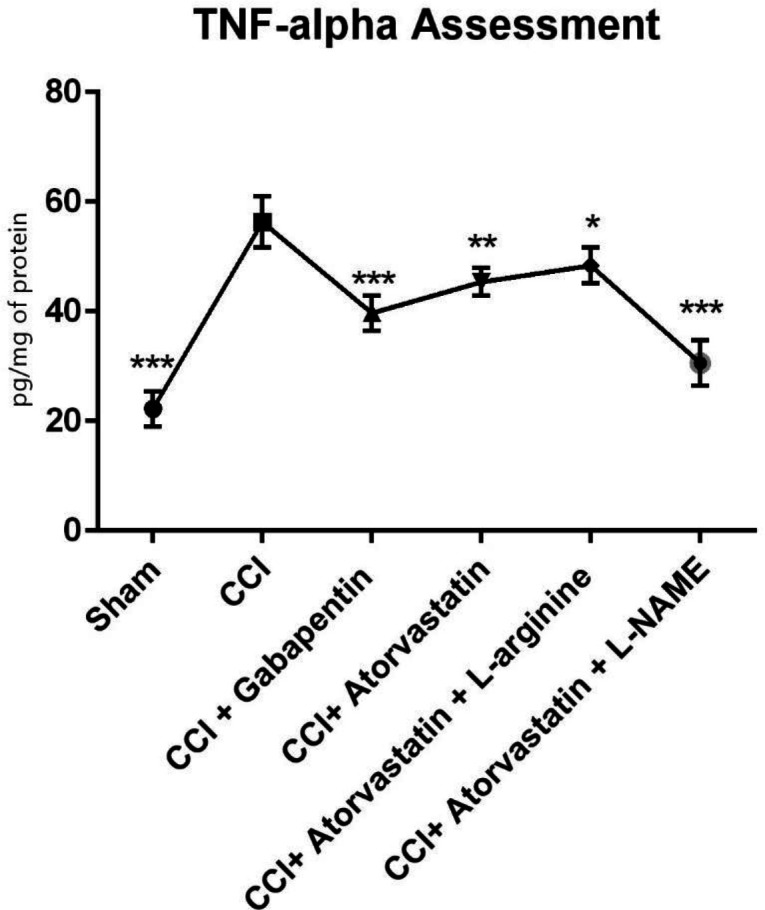
Effects of atorvastatin administration on CCI-induced rise in TNF-α level in the spinal nerve on fourteen days (n = eight rats per each group). ^*^P < 0.05, ^**^P < 0.01, ^***^P < 0.001 *vs.* CCI

**Figure 5:**
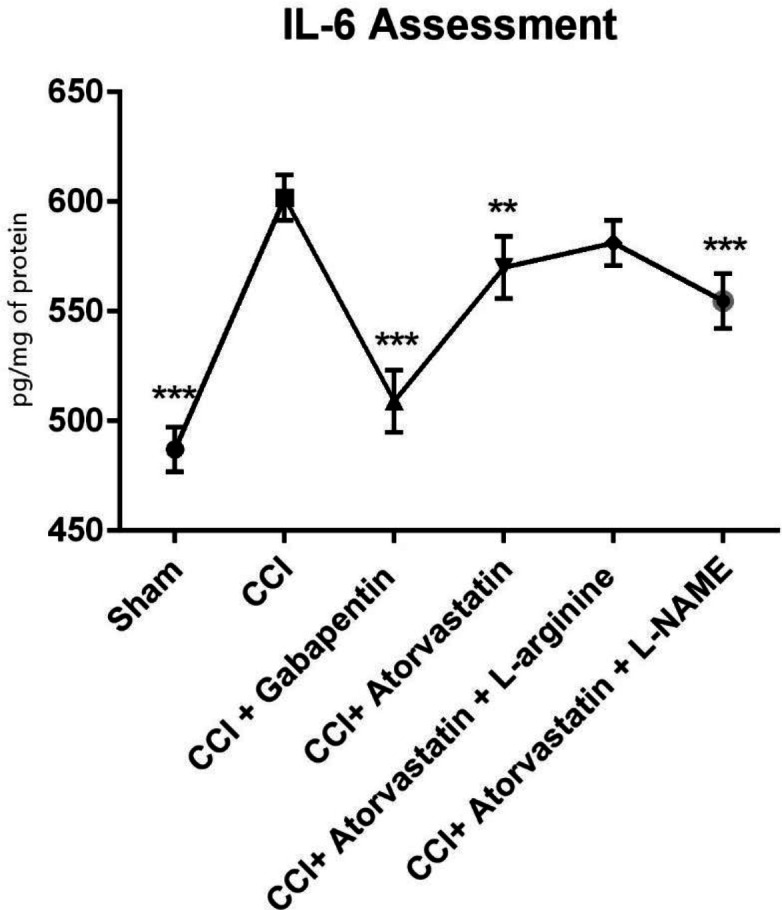
Effects of atorvastatin administration on CCI-induced rise in IL-6 level in the spinal nerve on fourteen days (n = eight rats per each group). ^*^P < 0.05, ^**^P < 0.01, ^***^P < 0.001 *vs. *CCI

As shown in Figure 4, the TNF-α level remarkably increased in the CCI-induced rats. However, the level of TNF-α was significantly decreased in the treated rats with atorvastatin and L-NAME (*P* < 0.001). In contrast, we found that in the CCI-rats treated with gabapentin, the level of TNF-α significantly decreased and remained at a normal level (*P* < 0.001). Furthermore, the levels of IL-6 increased in the second group 14 days after inducing NP in the rats by CCI (Figure 5). However, IL-6 notably decreased in the CCI + atorvastatin group (*P* < 0.01). Nevertheless, treatment with atorvastatin + L-NAME decreased the level of IL-6 to the normal level (*P* < 0.001) in the rats experienced with the CCI-induced NP. The level of this cytokine was found in its highest level in the CCI-rats treated with concomitant atorvastatin and L-arginine.

*Histological Evaluation in Sciatic Nerve*

Results showed that there was no sign of inflammation or morphological changes in the sciatic nerve among rats in the control groups (saline) as demonstrated. Moreover, morphological observations showed that there was extended perineural inflammation around the sciatic nerve in CCI (score = 3). Furthermore, the histopathological study demonstrated a low level of inflammation ratio around the sciatic nerve in the CCI rats treated with atorvastatin plus L-NAME (score = 1). The effect of atorvastatin + L-arginine on increased level of inflammation was statistically significant (score = 3).

## Discussion

In the present study, we found that the pain hypersensitivity to mechanical and thermal stimuli could persist for at least 2 weeks after CCI. However, treatment with atorvastatin prevented the decline in PWL, increased the allodynia score and prevented the development of hyperalgesia as well as hyperallodynia as compared to the CCI control. Our findings also revealed that treatment with L-NAME and atorvastatin, as well as treatment merely with atorvastatin significantly decreased the development of hyperalgesia and hyperallodynia and improved the mobility behaviors. In addition, the concurrent administration of atorvastatin with L-NAME has significantly prevented the perineural inflammation around the sciatic nerve and development of the hyperalgesia and allodynia, in comparison with the atorvastatin + L-arginine group. The CCI group exhibited the increased level of TNF_α and IL-6 in spinal nerve tissue. TNF_α and IL-6 levels were decreased in the group treated with atorvastatin and L-NAME. TNF_α levels were significantly improved in the CCI group treated with atorvastatin + L-arginine; however, the effect of atorvastatin plus L-arginine on IL-6 was not statistically significant. Inflammation around the nerve was statistically significant in the CCI rats treated with atorvastatin and L-NAME.

The CCI model is the most generally employed NP model of nerve injury-induced hyperalgesia and allodynia in animals (23). After peripheral nerve damage, neurotoxic chemicals and inhabitant immune cells, that gather around the injured area of the nerve, secrete proinflammatory cytokines (24). The rise in the levels of TNF-α, IL-6, and NF-ΚB occurs once nerve damage happens, and NP improves through reducing the release of these cytokines (25-27). In particular, TNF-α and IL-6 have been associated with causing pain in many animal models of neuropathic (28, 29). TNF-α has been described as a cytokine that plays a significant role in the “immune-to-brain” pathway of connection with pain (28). The classic CCI models in rats might exhibit high amounts of TNF-α in the hippocampus, locus coeruleus and red nucleus of CNS (30-32). Many investigations have indicated that IL-6 can be a major determinant in the nerve damage. It has also been associated with the initiation and preservation of NP (33, 34). A recent study has demonstrated that statins have the capacity to modify endothelial function, to decrease inflammatory reactions, and to reduce thrombus formation (35). Inflammatory cytokines were decreased after ATOR administratioin in mice (36). It has been indicated that atorvastatin protects the structural and functional integrity of nerves and preserves them against trauma and also, it attenuates NP (37, 38). In adittion, Barsante et el. have shown that the daily oral administration of ATOR effectively reversed the increase of inflammatory hypernociception (39). 

The functions of NO (synthesis from l-arginine) are regulated by the expression and activity of nNOS, eNOS, and iNOS (40). Neuronal NO synthases produce NO in the nervous system and have been shown to contribute to the spinal nociceptive. Furthermore, inducible nitric oxide synthases is involved in the mechanisms of central and peripheral sensitization in neuropathic pain and inflammation (41). Moreover, the high levels of NO are generated by iNOS over a longer period of time compared with the other nNOS and eNOS (42). It has been shown that NO production via nNOS and iNOS is involved in the maintenance of hypersensitivity (43). The observations indicate that the NO can up-regulate the TNFα expression and IL-6 (44, 45). L-NAME administration (non-selective inhibitor of NOS) is able to decreas the hyperalgesia and allodynia (46, 47). 

In summary, our results indicated that CCI could increase hyperalgesia and hyperallodynia. Furthermore, CCI apperedd to increase the levels of TNF-α and IL-6, pointing out the association between inflammation and NP. In addition, inhibiting NO by atorvastatin use ameliorates problems of NP in the experimental CCI model. Our results demonstrated that atorvastatin and L-NAME could attenuate hyperalgesia and allodynia. Moreover, they were able to decrease inflammation and at the same time they helped sciatic nerves to convalesce from histopathological scores.

## Conclusion

To put it briefly, this research proved that nitric oxide is able to play an efficient role in nociceptive signal processing during the major sensitization when the animals suffer from chronic constriction injury-induced neuropathic pain. Atorvastatin treatment (with inhibition of NO) alleviated the CCI-induced neuropathic pain and inflammatory cytokines, suggesting its prospective application in pain. 
